# AJAK inhibitor, tofacitinibreduces IL-6 and matrix metalloproteinase-3 productionin rheumatoid arthritis with suppressed cartilage destruction

**DOI:** 10.1186/ar3678

**Published:** 2012-02-09

**Authors:** Kunihiro Yamaoka, Satoshi Kubo, Koshiro Sonomoto, Keisuke Maeshima, Yoshiya Tanaka

**Affiliations:** 1 The First Department of Internal Medicine, University of Occupational and Environmental Health, Japan, Kitakyushu, Japan, 807-8555; 2 Department of Internal Medicine I, Faculty of Medicine, Oita University, Japan

## Background

Tofacitinib, targeting Janus kiase (JAK) has gained attention as anorally available new disease modifying anti-rheumatic drug with high clinical efficacy against rheumatoid arthritis (RA). While the clinical trial has progressed and the wide usage of tofacitinib is conceivable in the near future, the precise mechanism of action in RA patients remains to be solved.

## Materials and methods

Fifteen RA patients enrolled in tofacitinib clinical trial were randomized to 1, 3, 5 or 10 mg BID for 12 weeks. Serumwas collected at 0 and 12 weeks for further cytokine measurement by ELISA.To analyze the effect at the local inflammatory site, synovium and cartilage from a RA patient undergoing joint replacement was implanted to severe combined immunodeficiency (SCID) mice (SCID-huRAg mouse) andtofacitinib was administered via osmotic mini-pump and serological and histological investigation was performed.

## Results

Background of patients in clinical trial: mean age; 56.4 years, mean disease duration; 95.1 months, methotrexate (MTX) and tofacitinib were administered in all patients, median doses were 9.4 mg/week and 4.1 mg BID, glucocorticoids were administered in 6 patients, median dose was 5.4 mg/day. Baseline characteristics of the disease activity; SDAI 30.0, DAS28 (ESR) 6.3, HAQ 1.1, CRP 21.0 mg/l, ESR 57.1 mm/h, MMP-3 259.3 ng/ml, RF 216.2 U/ml. After 12 weeks treatment, disease activity decreased with statistical difference (p < 0.05) as follows; SDAI13.8, DAS28(ESR) 4.0, HAQ 0.8, CRP 8.1 mg/l, ESR 30.9 mm/h, MMP-3 149.9 ng/ml, RF 150.8 U/ml. Among the multiple cytokines measured, IL-6 and IL-8 tended to decrease, from 52.2 pg/ml to 28.2 pg/ml (p < 0.05) and from 41.7 pg/ml to 29.5 pg/ml (not significant), respectively. There was a statistically significant correlation between reduction of IL-6 and reduction of MMP-3.

In SCID-huRAg mouse, apparent invasion of RA-derived synoviuminto cartilage was observed, whileadministration of tofacitinibmarkedly suppressed invasion. In order to investigate the relevance with our findings from the patients in the clinical trial, cytokines in SCID-huRAg mouse serum was measured after administration of tofacitinib for 7 days. Interestingly, tofacitinib significantly decreased production of human IL-6 and IL-8 as well as human MMP-3 from 29.79 pg/ml to 2.89 pg/ml, 17.89 pg/ml to 4.22 pg/ml and 65.96 pg/ml to 33.13 pg/ml respectively.

## Conclusions

Tofacitinib improved disease activity and suppressed cartilage destruction with decreased serum IL-6 and IL-8 in both, RA patients and SCID-huRAg mouse in connection with reduced MMP-3. These results indicate that tofacitinib reduces inflammation by suppressing IL-6 production and consequently inhibiting cartilage destruction in the initial several months of administration.

